# Coseismic seafloor deformation in the trench region during the Mw8.8 Maule megathrust earthquake

**DOI:** 10.1038/srep45918

**Published:** 2017-04-05

**Authors:** A. Maksymowicz, C. D. Chadwell, J. Ruiz, A. M. Tréhu, E. Contreras-Reyes, W. Weinrebe, J. Díaz-Naveas, J. C. Gibson, P. Lonsdale, M. D. Tryon

**Affiliations:** 1 Departamento de Geofísica, Facultad de Ciencias Físicas y Matemáticas, Universidad de Chile, Santiago, Chile; 2 Scripps Institution of Oceanography, University of California, San Diego, CA, USA; 3 College of Earth, Ocean and Atmospheric Sciences, Oregon State University, Corvallis, OR, USA; 4 GEOMAR – Helmholtz-Centre for Ocean Research, Kiel, Germany; 5 Escuela de Ciencias del Mar, Pontificia Universidad Católica de Valparaíso, Valparaíso, Chile; 6 Lamont-Doherty Earth Observatory, Columbia University, New York, NY, USA

## Abstract

The *M*_w_ 8.8 megathrust earthquake that occurred on 27 February 2010 offshore the Maule region of central Chile triggered a destructive tsunami. Whether the earthquake rupture extended to the shallow part of the plate boundary near the trench remains controversial. The up-dip limit of rupture during large subduction zone earthquakes has important implications for tsunami generation and for the rheological behavior of the sedimentary prism in accretionary margins. However, in general, the slip models derived from tsunami wave modeling and seismological data are poorly constrained by direct seafloor geodetic observations. We difference swath bathymetric data acquired across the trench in 2008, 2011 and 2012 and find ~3–5 m of uplift of the seafloor landward of the deformation front, at the eastern edge of the trench. Modeling suggests this is compatible with slip extending seaward, at least, to within ~6 km of the deformation front. After the *M*_w_ 9.0 Tohoku-oki earthquake, this result for the Maule earthquake represents only the second time that repeated bathymetric data has been used to detect the deformation following megathrust earthquakes, providing methodological guidelines for this relatively inexpensive way of obtaining seafloor geodetic data across subduction zone.

Large slip near the Japanese trench has been reported for the 2011 Mw9.0 Tohoku earthquake by inversion of seismological data and tsunami waveforms^1^,^2^,^3^. This large near trench slip (up to 60 m) has been confirmed by the analysis of the seafloor deformation derived from GPS-Acoustic data^4^, direct submarine seafloor inspection^5^, changes in seafloor depth^6^ and sub-seafloor structure imaged on seismic reflection profiles acquired before and after the earthquake^7^. The northern Japanese subduction zone is generally thought to be an erosive margin characterized by thin sedimentary fill in the trench, and very small accretionary prism on the leading edge of the upper plate^8^, a scenario thought to allow slip to extend to the trench^9^. In contrast, margins characterized by thick sedimentary fill in the trench and a large accretionary prism is generally thought to be rheologically incompatible with stick-slip deformation^9^. Rupture along the up-dip subduction interface is expected to be dissipated (e.g., refs 10 and 11), however, joint inversions of GPS, teleseimic and tsunami data provided evidences of large co-seismic slip near the trench during the Maule earthquake^12^, and also during the Mw 7.8 Mentawai earthquake^13^, both located at accretionary margins. Accordingly, direct constraints of the up-dip limit of large earthquake rupture areas have important implications for the processes of tsunami generation and to understand the rheological behavior of the continental wedge.

The Maule earthquake occurred in a region of the Nazca-South America subduction zone where the trench is filled by ~1.5 s (~2 km) of sediment and the accretionary prism is 40–50 km wide^11^,^14^. The region of greatest coseismic slip during the Maule earthquake crosses the Chilean trench at ~34.5°S (refs 15, 16, 17, among others). Co-seismic slip models based on onshore geodetic and global and regional seismic data cannot resolve slip to the deformation front due to lack of constraints offshore (e.g., refs 17 and 18). However, addition of tsunami records seaward of the trench to the data for inversion of the slip history suggests slip to deformation front^12^ along the segment of the margin that we address with our study (black contours in Fig. 1) in spite of the presence of young, accretionary wedge sediments in the upper plate. Our analysis, in which we difference bathymetric data collected before and after the February 27, 2010 Mw 8.8 Maule earthquake, supports this conclusion by providing direct evidence for a change in the elevation of the continental wedge near the deformation front.

## Results

**Figure 1:**
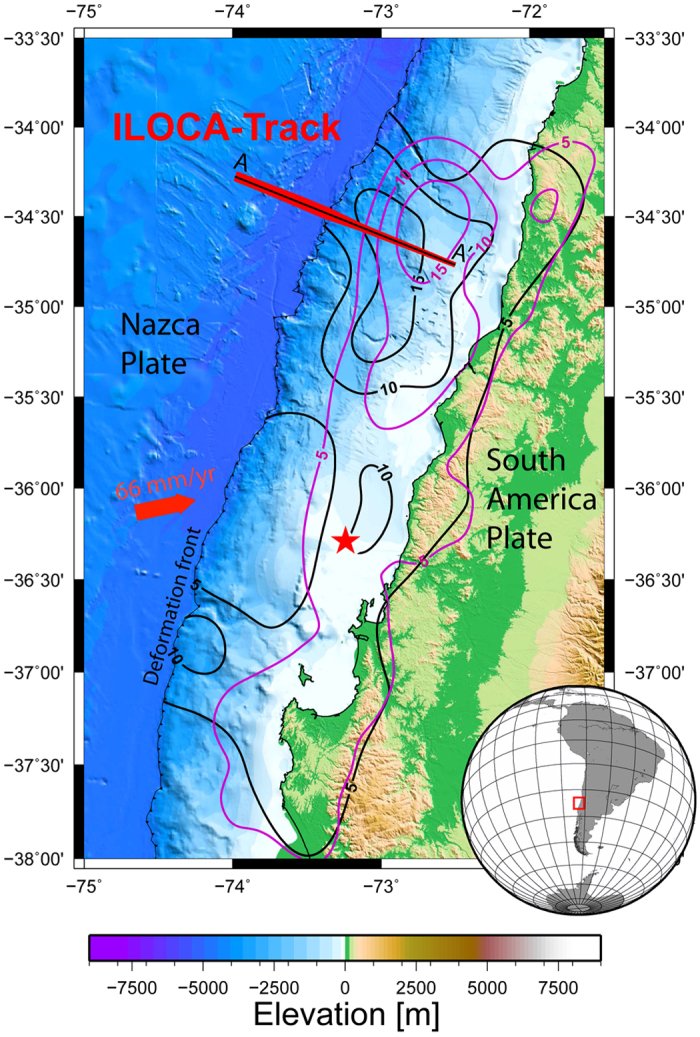
Map of the study area. A-A’ corresponds to the bathymetric ILOCA track. Colored grid shows the elevation in the area. Red star is the epicenter of the 2010 M_w_ 8.8 Maule earthquake. Magenta lines are the iso-contours of slip during the Maule earthquake according to Tong *et al*.^15^ and black lines are the iso-contours of slip obtained by Yue *et al*.^12^. Maps in the figure were created using GMT (Generic Mapping Tools, http://gmt.soest.hawaii.edu/) software.

**Figure 2:**
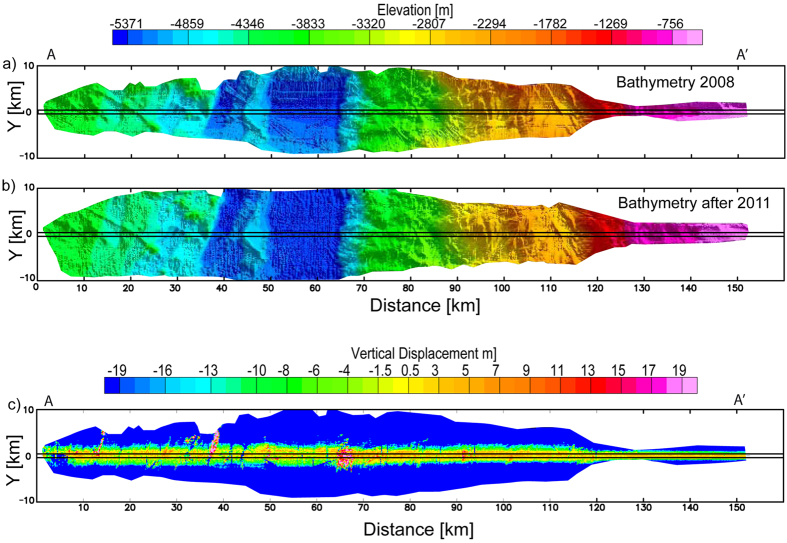
Bathymetric grids along the ILOCA Track. (**a**) Bathymetry acquired in 2008. (**b**) Bathymetry acquired in 2011 and 2012. (**c**) Elevation change obtained by the subtraction of the grid shown in A from the data shown in B. The black rectangle in the center of the track includes the data that are analyzed.

**Figure 3:**
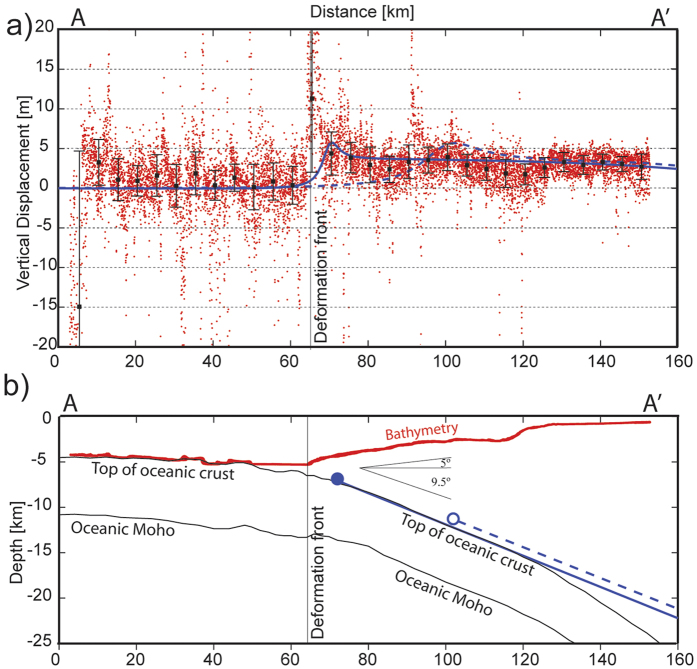
Data projected to the central line of ILOCA track. (**a**) Red dots are the elevation change in each node of the bathymetric grids. Black squares correspond to maximum data densities computed in several bins along the profile. Blue line indicates the vertical displacement of the preferred dislocation model, while dashed blue line corresponds to the model with a deeper up-dip. (**b**) Subduction zone along the ILOCA track. Blue line and blue dot indicate the fault plane and the up-dip of our preferred dislocation model, while dashed blue line and white dot correspond to the model with a deeper up-dip.

Multi-beam bathymetric data were collected in 2008 as part of a geophysical survey along a track crossing the subsequent region of maximum uplift (ILOCA, Fig. 1). This track was remapped following the earthquake in 2011 and again in 2012. Seafloor depths (Fig. 2a,b) were calculated using MB-Systems^19^ software to ray-trace the swath-mapper multi-beam sonar soundings through four seawater sound speed profiles collected evenly along the ILOCA track in 2011 and 2012 (See Methods in supplementary material). The seafloor soundings were gridded at 100 m spacing. Depth and positioning errors inherent in the outer beams were avoided by including only data within a narrow region around the nadir (Fig. 2c; ref. 20). Assuming depth changes parallel to the margin are small within this narrow region, these data were projected onto profile A-A‘ at the center of the ILOCA track (red dots in Fig. 3a).

The elevation differences were then divided into a sequence of segments along the ILOCA profile. Within each segment, a histogram was formed and a scaled T-student probability density function was used to calculate the mean elevation and its variance (black squares in Fig. 3a). The mean elevation changes reveal that the seafloor landward of the deformation front was uplifted by 3–5 m relative to the seafloor seaward of the trench.

To test whether the observed uplift is consistent with slip on the plate boundary during 2010 Mw 8.8 Maule earthquake, we calculated the deformation generated by a simple dislocation model in an elastic half-space^21^ assuming for a slab dip of 9.5°, consistent with seismic refraction data, and a free surface with a dip of −5°, based on bathymetry (see Fig. 3b). We assumed a Poisson’s ratio of 0.25, a fault size of 150 km width and 450 km length, and a homogeneous pure reverse slip of 12 m. We analyzed two models where the plane fault extends to within 6 km of the deformation front in the first case (blue dot in Fig. 3b) and to within 40 km of the deformation front in the second case (white circle in Fig. 3b). The vertical deformation calculated for the first case matches the observed elevation change near the trench while the model for the second case predicts nearly zero vertical deformation within the region 20 km landward of the deformation front. The simple elastic model with slip to within 6 km of the deformation front is therefore, compatible with the observed elevation changes.

**Figure 4:**
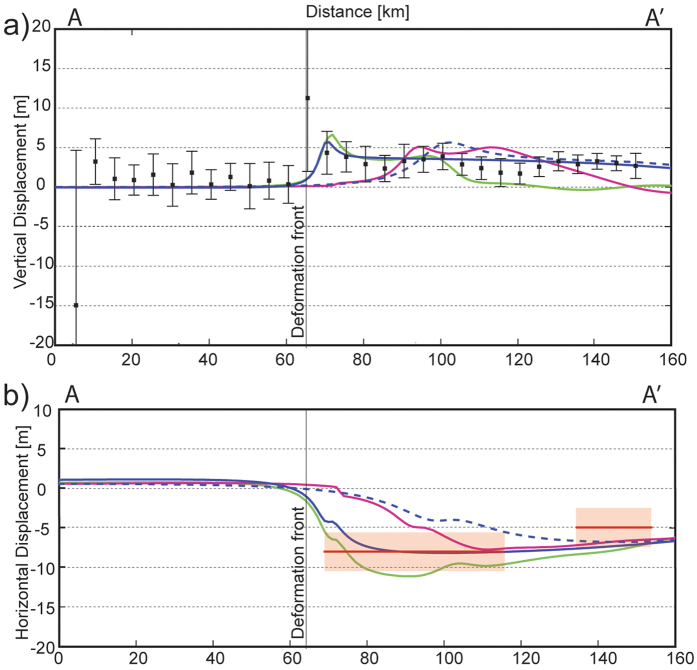
Models of vertical and horizontal displacements along the ILOCA Track. (**a**) Black squares are the same as in Fig. 3a. Blue line and dashed blue line are the same as in Fig. 3a. Green line and magenta line are the vertical displacements calculated using the slip models of Yue *et al*.^12^ and Tong *et al*.^15^ respectively. (**b**) Horizontal displacement corresponding to the same models presented in (**a**). Red lines are the horizontal displacement derived by L1 norm minimizing strategy (the approximated error bars are indicated in light red).

In Fig. 4, we compare the observed uplift to the deformation predicted along A-A’ by two published co-seismic slip models (Fig. 1). The model of Tong *et al*.^15^ is representative of several slip solutions that have a high slip patch near 34.5°S but low slip near the trench (magenta iso-slip contours in Fig. 1). In contrast, the model of Yue *et al*.^12^ places the high slip patch farther west (black iso-slip contours in Fig. 1). The deformation along A-A’ corresponding to the Yue *et al*. model^12^ (green in Fig. 4a) better matches the seafloor elevation change than that calculated for the Tong *et al*. model^15^ (magenta line in Fig. 4a).

**Figure 5:**
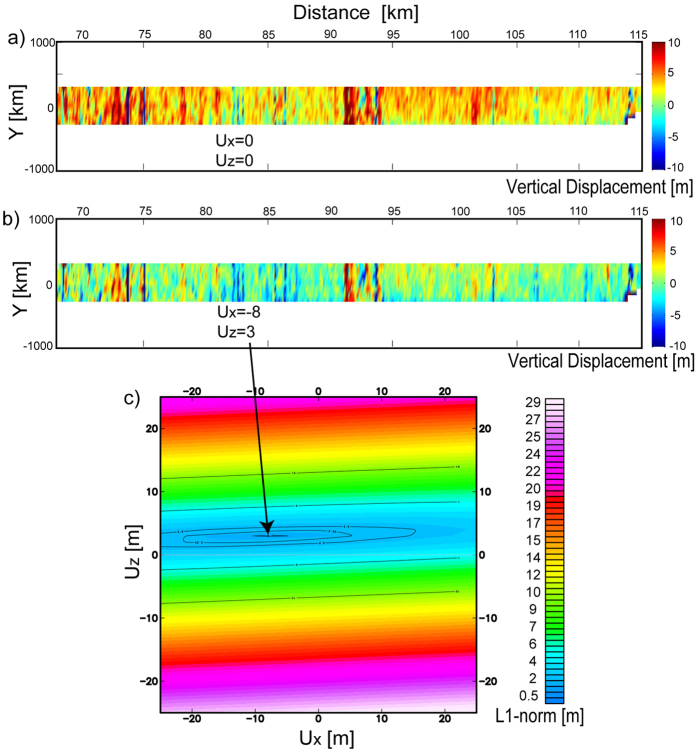
Estimation of vertical and horizontal deformation by the differences minimization. (**a**) Direct subtraction of 2008 data from 2011–2012 data. (**b**) Subtraction of 2008 data from a displaced grid of 2011–2012 data. The values of horizontal (along the track) and vertical displacements are dx = −8 m and dz = 3 m respectively. (**c**) Mapping of L1-norm of the differences between the post-earthquake bathymetry displaced in the space and the bathymetry before the seismic event. The minimum value in is indicated by the black arrow.

We attempted to estimate horizontal as well as vertical motion of the seafloor by minimizing the L1 norm of the difference between the pre-earthquake and post-earthquake bathymetry to simultaneously solve for uniform horizontal (ux) and vertical (uz) displacements of the post-earthquake data along profile A-A’^6^,^20^. Landward of the deformation front in a sector defined between 68 km < x < 115 km we observed values of u_x_ = −8 m, u_z_ = 3 m, while in a sector defined between 134 km < x < 160 km, u_x_ = −5 m and u_z_ = 3 m. A negative value of u_x_ corresponds to a seaward displacement of the seafloor during the earthquake, and a positive value of u_z_ is an upward displacement of the seafloor. Within the western sector, the u_x_ values are in agreement with the horizontal displacements predicted by our simple model (which consider 12 m slip to within 6 km of the deformation front, blue line in Fig. 4b) and with the horizontal displacements predicted by Yue *et al*.^12^ (green line in Fig. 4b). However, the u_x_ estimate has large uncertainty (Fig. 5), probably related to the low sentivity of the bathymetric soundings to the horizontal seafloor displacement, particularly using near-nadir data in zones with regional small slope angle of the bathymetry, ref. 22. Nevertheless, this technique present high confidence for the vertical displacement estimation (Fig. 5), which confirms a coseismic uplift of about 3 m using an approach independent to the statistical analysis presented earlier (see details in Methods and supplementary material).

## Discussion

Our results is consistent with the inference of Yue *et al*.^12^, who obtained co-seismic slip extended to near the trench along this segment of the margin. By the interpretation of the bathymetric data with a simple Okada model, we can conclude that rupture associated to the Maule earthquake affect, at least, to within ~6 km of the deformation front (see Fig. 3). Models with the up-dip closer to the deformation front also can explain the data, in particular the apparent increase of the uplift immediately landward from the deformation front. However, as is showed by the statistical analysis, the standard deviation of the data in that zone (error bars in Fig. 3a) is larger than that obtained for the rest of the profile, which prevents and exact interpretation in that segment of the profile. In fact, similar picks are observed in zones where the bathymetric gradient changes rapidly (associated for instance with the horst-graven structures in the oceanic plate), suggesting that the transition from the flat seafloor at the trench to the lower continental slope can increase the noise in the swath-bathymetry data. On the other hand, reflection seismic profiles acquired along the ILOCA track before and after the earthquake (see supplementary material), do not show evidence of new deformation inside the trench and/or around the deformation front, which shows that under the limitations of the seismic method, the trench where not affected during the earthquake. Then, according our information, the up-dip of the Maule earthquake was located between the deformation front and ~6 km landward.

In order to define the properties of the interplate boundary near the trench, it is important to investigate if the observed uplift was co-seismic or whether it can represent post-seismic stress relaxation. The observed seafloor deformation is potentially the combination of coseismic motion, afterslip, visco-elastic relaxation and fault relocking. After-slip occurs when the rupture zone slips in the same direction as the main event and decays rapidly over time^23^. This process has been explained in the context of the velocity strengthening behavior^24^, and has been observed mainly in the down-dip region of megathrust-earthquakes^25^,^26^. At longer time scales, post-seismic deformation can be modeled as viscoelastic relaxation of the subducted slab, underlying asthenosphere and overlying asthenospheric mantle wedge^23^,^27^,^28^. Viscoelastic relaxation models predict post-seismic deformation observed for several years or decades after the earthquake, including subsidence over the down-dip limit of co-seismic slip and on the rupture area and uplift seaward^29^. The effect of relocking is likely minimal the first few years following a large event^30^.

Due to the scarcity of offshore observations, no observations of the post-seismic deformation in the up-dip zone of the megathrust earthquakes are available for most events. For the Maule earthquake, Bedford *et al*.^26^ observed ~0.2 m of post-seismic subsidence above the down-dip limit of co-seismic slip in the first year after the earthquake, which can be explained by after-slip. Hsu *et al*.^31^ observed 0.17 m of subsidence near the up-dip limit of slip during the 2005 Mw8.7 Nias-Simeulue earthquake in the first year after the event, which is also explained by after-slip. On the other hand, Watanabe *et al*.^23^ show values of 0.14–0.45 m of subsidence over the rupture area of the Tohoku earthquake during the first three years after the earthquake, and explain their observations by the viscoelastic relaxation process. Therefore, independently of the physical mechanism involved, previous studies suggest a maximum of about ~0.5 m of post-seismic change in elevation in the two years after a large earthquake, which is a small fraction of the ~3–5 m of uplift observed in our bathymetric analysis. Consistent with the observations, published models of post-seismic deformation in the Nias-Simeulue and Maule earthquakes regions show after-slip maxima that are less than 2.5 m during the first year after the main event^26^,^31^,^32^, which is a factor of 5 lower than the ~12 m necessary to explain the uplift detected by our bathymetric analysis (Figs 3 and 4).

The evidence discussed above suggests that most of the deformation that we observe was generated during the Maule earthquake. However, it is difficult to ensure that the deformation reached the zone near the trench during the co-seismic rupture, because the post-seismic bathymetry was acquired several months after the earthquake. Then, the observed deformation for Maule earthquake could be generated during the co-seismic rupture but also in a short period after the earthquake (clearly before 1 year after the main event).

Why the rupture propagated updip of the normal seismogenic zone is not fully understood. In general, one would expect the accretionary prism adjacent to the deformation front, which is thought to be comprised of poorly consolidated material, to have a velocity strengthening behavior. However, if the base of the accretionary prism is in a conditionally stable frictional regime, velocity weakening behavior can be induced by high rupture velocity due to an earthquake nucleated in a deeper unstable zone^9^. Considering the case where the observed deformation is co-seismic, the Maule earthquake rupture is consistent with this mechanism because the main zone of near-trench slip is located immediately adjacent to the highest co-seismic slip patch^12^.

The outermost portion of the wedge (the frontal zone of the accretionary prism) is comprised of poorly consolidated material that should have a velocity strengthening behavior. In that case, the friction on the plate boundary increases during the earthquake, which stopped the rupture propagation and generated co-seismic compressional elastic (or brittle) deformation inside the wedge^24^. This could be why, according to the seismic reflection data, the brittle rupture of the Maule earthquake did not reach the trench, in contrast to trench-breaching slip during the Tohoku earthquake (see seismic reflection profile along A-A’ in the supplementary material).

The result indicates that slip can occur near the trench in accretionary margins as well as in an erosive margin, like the northern Japanese subduction zone. Our conclusion that slips extended, at least, to ~6 km landward of the deformation front along profile A-A’ is consistent with the inference of Yue *et al*.^12^, who showed slip extended to the trench in the northern portion of the Maule earthquake rupture (northward of ~35°S). According the same authors^12^, a second local patch of high slip was extended near the trench in the southern portion of the rupture (~37°S, see Fig. 1), which suggest the mechanical behavior observed at ILOCA track could be an usual feature in this zone of the margin. This has important implications for seismic hazard evaluation and tsunami sources studies, because some regions of the interplate boundary beneath the accretionary prism can be activated by an earthquake nucleated in depth and others regions can be deformed aseismically during the seismic cycle. Therefore, a new challenge for seismological studies is to characterize the parameters that control the stable or critically stable behavior along the uppermost portion of subduction zone.

Due to the high costs of operation, only few stations for seafloor GPS-Acoustic observations can be installed to study large portions of subduction zones. Therefore, the repeating bathymetry method represents a complementary technique to cover large portions of the margin with enough resolution to study the seafloor deformation during the seismic events, at least for large earthquakes. For this reason, our result highlights the importance of systematic acquisition of high-quality bathymetric data along seismic gaps in convergent margins to compare with post-earthquake measurements, in order to have a better understanding of the co-seismic and post-seismic seafloor deformation.

## Methods

Two years before the Maule earthquake (in March, 2008), the ILOCA track was acquired by the R/V RSS James Cook under the project SBF 574 of IFM-GEOMAR. After the earthquake, the same bathymetric track was acquired in March 2011 and in May 2012 by the R/V Melville during the expeditions of the NSF projects Chilean Earthquake Rupture Survey and ChilePEPPER. Before and after the main event, there are pairs of tracks that were navigated in opposite directions (NW-SE and SE-NW), which after joint processing of the data, allows to decrease the pitch and roll effects on the resulting processed bathymetry. In 2008, the ILOCA track was surveyed three times using a Kongsberg EM120 12 kHz multibeam echo; twice in the direction from southeast to northwest (seaward) and once from northwest to southeast (landward). The vessel speed was nominally 4–6 kts for all three surveys. In 2011, using the EM122 multibeam echo sounder, two swaths were collected over this same track; one going seaward and one landward at a nominal speed of 12 kts. In 2012, the EM122 aboard R/V Melville was again used to collect one swath in the seaward direction at nominally 12 kts. The processing of the multibeam bathymetric data was performed using the MB-System software package. Manipulations of the bathymetric grids were implemented with MATLAB routines. Data visualizations were made by using OasisMontaj and GMT software.

The Kongsberg EM120 and EM122 share the same acoustic hardware and bandwidth, with the EM122 providing upgraded signal processing capabilities. MB-System was used to remove erroneous soundings first through automatic editing which involves systematic flagging of beams outside of ranges set by the user. For our purposes along track beams were flagged if they exceeded ±20% (±1000 m in 5000 m depth) of the median depth while across track beams were flagged if they were a distance less than 1% or more than 20% of the median depth from adjacent beams (50 m/1000 m in 5000 m depth). Additionally, beams were automatically flagged if the across track beam to beam angle exceeded 45**°.** The individual pings were then manually edited in order to flag any beams that were erroneous, but not detected using the automatic method. All data were then tide corrected using the MB-System variant of the OTPS2 tide correction software, which combines a low-resolution global model with a high-resolution local model. Next, to ensure consistent calculation of seafloor depths a composite sound speed structure was compiled along the ILOCA track. Four expendable bathythermographs (XBT) profiles collected along the track in 2012 were combined with a surface to seafloor conductivity, temperature and depth (CTD) profile collected midway along the track in 2011 (Figure S1, see supplementary information). Finally, the three surveys from 2008 cruise were combined and gridded at 100 m spacing to form the pre-earthquake bathymetric profile. The two surveys in 2011 were combined with the survey in 2012 and gridded at 100 m spacing to form the post-earthquake bathymetric profile.

As was mentioned in the main text, the direct difference between the bathymetry, before and after the earthquake, shows that only a central band of the data has physical meaning. An increase of errors from the center of the track to the borders is inherent to the multibeam technique. In particular, an error in the beam angle (referred to the vertical direction below the vessel) generates a minimum error in the calculated depth at the center of the track, but its effects increase rapidly in function to the beam angle^33^. On the other hand, An analysis of the processed bathymetric grids showed that a static depth-shift of −1.71 m is observed between the post-earthquake grid and pre-earthquake grid in the zone of the profile extended seaward from the deformation front, which is assumed not be deformed by the earthquake. This static depth-shift is probably due to the different ambient conditions presents during the data acquisition, and then, it is considered as a systematic error that should be removed from that data before the analysis and interpretations. All the results presented here are calculated after this shift removal.

In order to highlight the bathymetric changes after the Maule earthquake, we performed a statistical analysis of the data corresponding to elevation change at every node along the central band of the ILOCA track (red points in Fig. 3a of the main text). The procedure started with the splitting of the data in 30 segments along the track, each one being 5 km wide. Then, using Matlab tools, we generated a histogram for the data in a specific segment, considering 20 bins to divide the range of the elevation change (u_z_) observed in that segment (see Figure S2 in the supplementary information). Finally, we fit a probability density function (PDF) to represent the shape and the amplitude of the histogram. To take account shape observed in the histograms, we applied a t-Student Location-Scale distribution. The maxima of the histograms are well represented by this PDF. We estimated the mean and standard deviation of the data with the t-Student Location-Scale, and the results correspond to the black squares and the associated error bars in Figures 3a and 4a of the main text.

Additionally to the statistical analysis of the data, we estimate the horizontal and vertical deformation during the Maule earthquake performing a calculation based on a simple assumption. If the shape of the seafloor remains approximately constant during the earthquake, at regional scale, the deformation measured can be seen as a horizontal (perpendicular to the trench) and a vertical displacement of the same seafloor shape, i.e, a translation without internal deformation. Therefore, if we know these horizontal and vertical displacements, we can restore the bathymetry acquired after the earthquake to its original position (before the earthquake), and then, to compare this “corrected” bathymetry with the bathymetry acquired before the earthquake. We can use, for instance, a L1 norm to compare the bathymetries obtaining a value near zero if the horizontal and vertical displacement applied are corrects. Based on this simple criterion, we calculated both displacement values (u_x_, u_z_). The result of this L1 norm minimization shows a clear uplift of 3 m during the earthquake, landward from the deformation front and an almost null vertical deformation seaward from the deformation front. Considering that this technique is highly dependent on u_z_ variations (see details in the supplementary information), the result confirms the vertical deformation pattern obtained by the statistical analysis using a completely different approach (see Fig. 5 in the main text and supplementary information).

## Additional Information

**How to cite this article**: Maksymowicz, A. *et al*. Coseismic seafloor deformation in the trench region during the Mw8.8 Maule megathrust earthquake. *Sci. Rep.* **7**, 45918; doi: 10.1038/srep45918 (2017).

**Publisher's note:** Springer Nature remains neutral with regard to jurisdictional claims in published maps and institutional affiliations.

**Publisher's note:** Springer Nature remains neutral with regard to jurisdictional claims in published maps and institutional affiliations.

## Supplementary Material

Supplementary Material
