# Supplementary material for: Coseismic seafloor deformation in the trench region during the Mw8.8 Maule megathrust earthquake
Source: Sci Rep. 2017 Apr 5;7:45918. doi: 10.1038/srep45918 (PMC5381107; doi:10.1038/srep45918)
Supplement: Supplementary Material [file srep45918-s1.pdf]

Supplementary Information

Coseismic seafloor deformation in the trench region during the Mw8.8 Maule megathrust earthquake

Authors: A. Maksymowicz, D. Chadwell, J. Ruiz, A. M. Tréhu, E. Contreras-Reyes, W. Weinrebe, J. Díaz-Naveas, J. C. Gibson, P. Lonsdale, and M. D. Tryon

Materials and Methods:

Two years before the Maule earthquake (in March, 2008), the ILOCA track was acquired by the R/V RSS James Cook under the project SBF 574 of IFM-GEOMAR. After the earthquake, the same bathymetric track was acquired in March 2011 and in May 2012 by the R/V Melville during the expeditions of the NSF projects Chilean Earthquake Rupture Survey and ChilePEPPER . Before and after the main event, there are pairs of tracks that were navigated in opposite directions (NW-SE and SE-NW), which after joint processing of the data, allows to decrease the pitch and roll effects on the resulting processed bathymetry. The analyzed bathymetric grids were generated with a cell-size of 100 m x100 m. The processing of the multibeam bathymetric data was performed using the MB-System software package. Manipulations of the bathymetric grids were implemented with MATLAB routines. Data visualizations were made by using OasisMontaj and GMT software.

As was mentioned in the main text, the direct difference between the bathymetry, after and before the earthquake (Figure 2c), shows that only a central band of the data has physical sense with values ranged in the ±10 m interval. An increase of errors from the center of the track to the borders is inherent to the multibeam technique. In particular, an error in the beam angle (referred to the vertical direction below the vessel) generates a minimum error in the calculated depth at the center of the track, but its effects increase rapidly in function to the beam angle (Hare, 1995). Then, the selection of the data in the center of the track is a reasonable way to reduce the uncertainties of the deformation analysis. A simple analysis of the processed bathymetric grids showed that a static depth-shift of -1.7051m is observed between the post-earthquake grid and pre-earthquake grid in the zone of the profile extended seaward from the deformation front, which is assumed not be deformed by the earthquake. This static depth-shift is probably due to the different ambient conditions presents during the data acquisition, and then, it is considered as a systematic error that should be removed from that data before the analysis and interpretations. All the results presented here are calculated after this shift removal.

In order to highlight the bathymetric changes after the Maule earthquake, we performed a statistical analysis of the data corresponding to elevation change at every node along the central band of the ILOCA track. The procedure started with the division of the data in 30 segments

along the track, each one with 5 km wide. Then, using Matlab tools, we generated a histogram for the data in a specific segment, and finally, we fit a probability density function (PDF) to represent the shape and the amplitude of the histogram.

Multibeam Data Processing

Within three weeks following the 27 Feb. 2010 Maule Earthquake an NSF-sponsored Rapid-Response Cruise (MV2010) used the Kongsberg EM122 12 kHz multibeam echo sounder aboard the R/V Melville to collect a swath bathymetric map of the entire rupture region. This was compared with bathymetric map of the region prior to earthquake composed of surveys from a variety of sources. The purpose was to look for any sizeable landslides that could have occurred due to the earthquake. However, no new landslide scars were found. Several bottom pressure sensors were also deployed, for recovery in 2011. A close examination of the swath bathymetric collected prior to 2008 revealed one track (referred as ILOCA track) perpendicular to the trench that extended 155 km SE of the across the continental slope in the region of maximum uplift as determined by land-based geodesy and regional and global seismic records.

In March 2008, the ILOCA track was surveyed three times using a Kongsberg EM120 12kHz multibeam echo sounder aboard the RRS John Cook; twice in the direction from southeast to northwest (seaward) and once from northwest to southeast (landward). The vessel speed was nominally 4-6 kts for all three surveys. In March 2011, using the EM122 multibeam echo sounder aboard the R/V Melville, two swaths were collected over this same track; one going seaward and one landward at a nominal speed of 12 kts. In May 2012, the EM122 aboard R/V Melville was again used to collect one swath in the seaward direction at nominally 12 kts.

The acoustic soundings from all six surveys of the ILOCA track were reprocessed in a consistent manner starting from the original raw data files. The Kongsberg EM120 and EM122 share the same acoustic hardware and bandwidth, with the EM122 providing upgraded signal processing capabilities. MB-System was used to remove erroneous soundings first through automatic editing which involves systematic flagging of beams outside of ranges set by the user. For our purposes along track beams were flagged if they exceeded ±20% (± 1000m in 5000m depth) of the median depth while across track beams were flagged if they were a distance less than 1% or more than 20% of the median depth from adjacent beams (50m/1000m in 5000m depth). Additionally, beams were automatically flagged if the across track beam to beam angle exceeded 45°. The individual pings were then manually edited in order to flag any beams that were erroneous, but not detected using the automatic method. All data were then tide corrected using the MB-System variant of the OTPS2 tide correction software, which combines a low-resolution global model with a high-resolution local model. Next, to ensure consistent calculation of seafloor depths a composite sound speed structure was compiled along the ILOCA track. Four expendable bathythermographs (XBT) profiles collected along the track in 2012 were combined with a surface to seafloor conductivity, temperature and depth (CTD) profile collected midway along the track in 2011 (Figure S1). No nearby XBT or CTD profiles were collected during the 2008 RRS James Cook cruise. A comparison of two adjacent XBT profiles from 2011 and 2012 showed no significant differences between data collected 15 months apart. Soundings from each

of the six surveys were retraced using the profile that was nearest. This essentially partitions the sound speed into four regimes along the ILOCA track.

Additionally, having surveys along the track in opposite directions both before and after the earthquake allowed estimation of a possible bias due to pitch in the multibeam echo sounders on both the RRS James Cook and the R/V Melville. A pitch bias could cause an elevation offset in sloped regions where the seafloor transitions from flat to sloped, e.g., between the flat seafloor west of the deformation front and the sloped seafloor to the east. No pitch bias was found in either system. Finally, the three surveys from the March 2008 RRS James Cook cruise were combined and gridded at 100 m spacing to form the pre-earthquake bathymetric profile. The two R/V Melville surveys in 2011 were combined with the survey in 2012 and gridded at 100 m spacing to form the post-earthquake bathymetric profile.

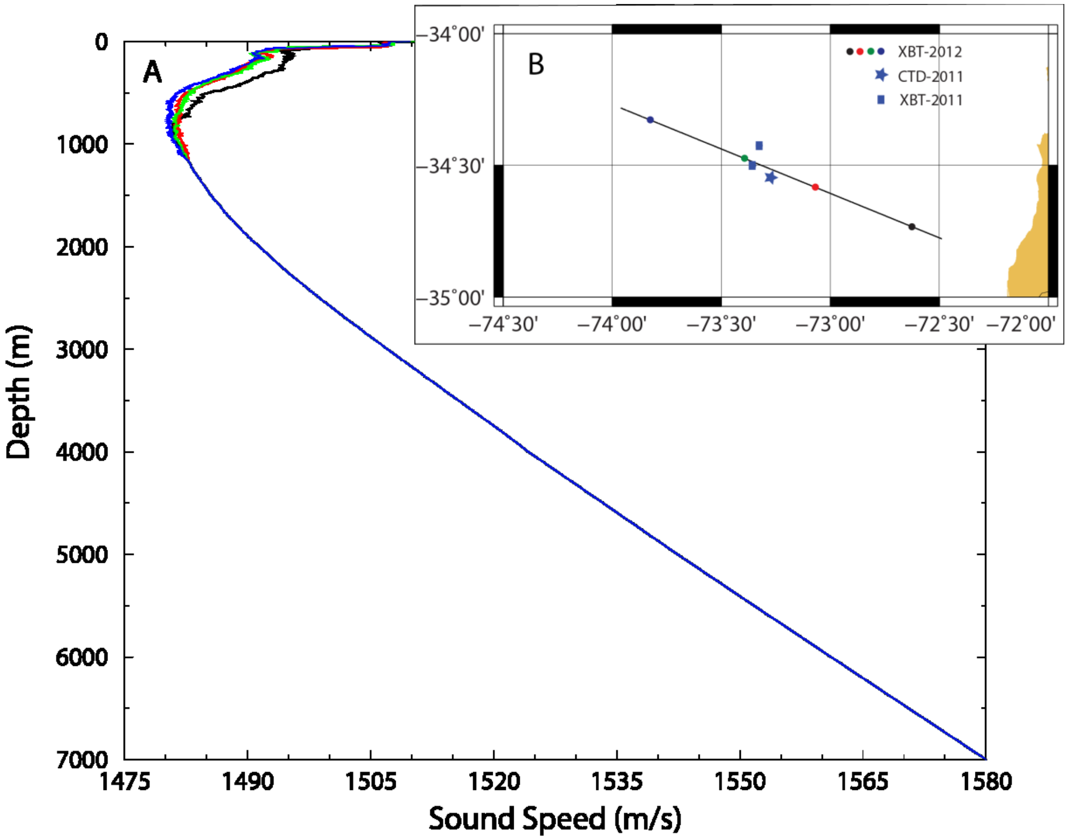

Figure S1 | Sound speed data. **a**, Four sound speed profiles used for the ray-tracing of all soundings. **b**, location of XBT and CTD profiles along the ILOCA track.

Statistical analysis of the elevation changes

In order to highlight the bathymetric changes after the Maule earthquake, we performed a statistical analysis of the data corresponding to elevation change at every node along the central band of the ILOCA track (red points in Figure 3b of the main text). The procedure started with the splitting of the data in 30 segments along the track, each one being 5 km wide. Then, using Matlab tools, we generated a histogram for the data in a specific segment, considering 20 bins to divide the range of the elevation change (Uz) observed in that segment (see Figure S2). Finally, we fit a probability density function (PDF) to represent the shape and the amplitude of the histogram.

In Figure S2, we present the fit of three different PDF for each analyzed segment. As we can see, the maximums of the histograms are well constrained with the Normal (or Gaussian) distribution (magenta lines in Figure S2). Analyzing the Normal distributions, it is clear that for the segments seaward of the deformation front (i.e. distances < 60 km along the profile), the maximum is located around Uz = 0 m, which suggests no deformation, but for the segments located landward from the deformation front (x > 60 km), the maximum is near Uz = 3 m, i.e., the means of the data show vertical deformation. However, the Normal distribution is not able to reflect the correct decay of the histograms around the maximum, which is in general more abrupt; the associated standard deviation is overestimated with this PDF.

To take account the decays observed in the data, we applied a t-Student Location-Scale distribution (cyan lines in Figure S2). The maxima of the histograms are well represented by this PDF, and the decays fit better than in the case of Normal PDF. We estimated the mean and standard deviation of the data with the t-Student Location-Scale, and the results correspond to the black squares and the associated error bars in Figure 3b of the main text.

The t-Student Location-Scale and the Normal are symmetric PDFs, but some of the histograms show an asymmetric shape. For this reason we also fit a Generalized Extreme Value distribution that merge the behaviors of the Gumbel, Weibull and Frechet asymmetric PDF (see MathWorks reference page, http://www.mathworks.com/). The result (blue lines in Figure S1) shows that this PDF works well for the histograms with highly asymmetric shape (see x=5.5 km and x=65.5 km), but in general, the amplitude of the maximum is not resolved correctly and the decays have the same problems that the observed for the Normal PDF. Then, for simplicity, we preferred to use the symmetric t-Student Location-Scale to analyze the amplitude and shape of the main lobe of the histograms that are approximately symmetric.

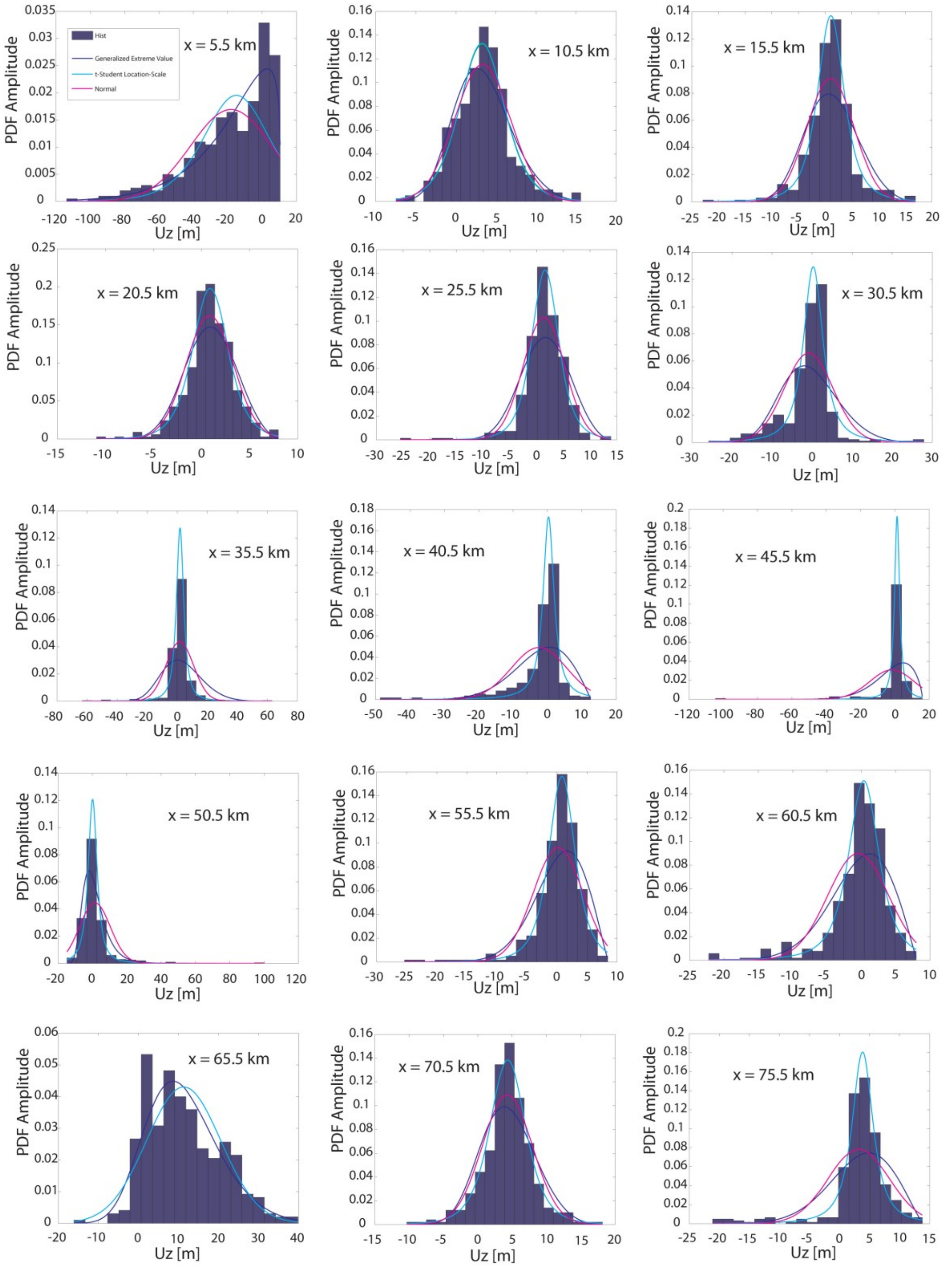

Figure S2 | Histograms of the 30 segments defined along the ILOCA track and the modeled Probability Density Functions. The corresponding segment is identified with the position of its center along the track (x). Magenta line corresponds to the Normal distribution fitted to the histogram, cyan line is the t-Student Location-Scale distribution fitted to the histogram, and the blue line shows the Generalized Extreme Value distribution fit.

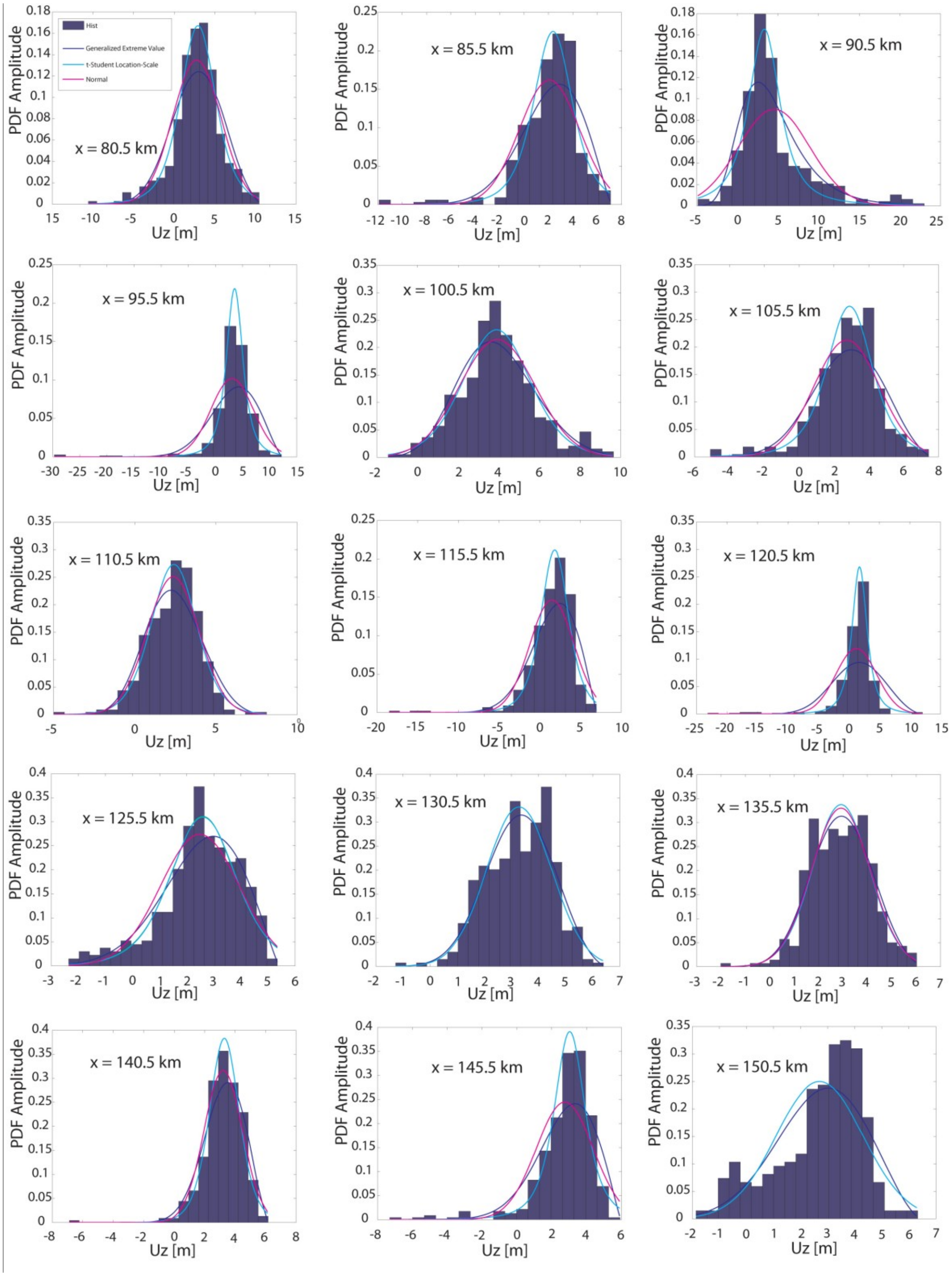

Figure S2 (continued)

In Figure S3, we present the calculated means and standard deviations obtained along the ILOCA track for the three PDF analyzed. As we can see, despite the high standard deviation associated to Normal and Generalized Extreme Value Distribution, the three PDFs highlight a bathymetric change landward from the deformation front. Our preferred PDF (t-Student Location-Scale) shows a similar standard deviation for all data along the track, which supports the direct comparison between all data, and supports the interpretation of the vertical deformation landward from the deformation front.

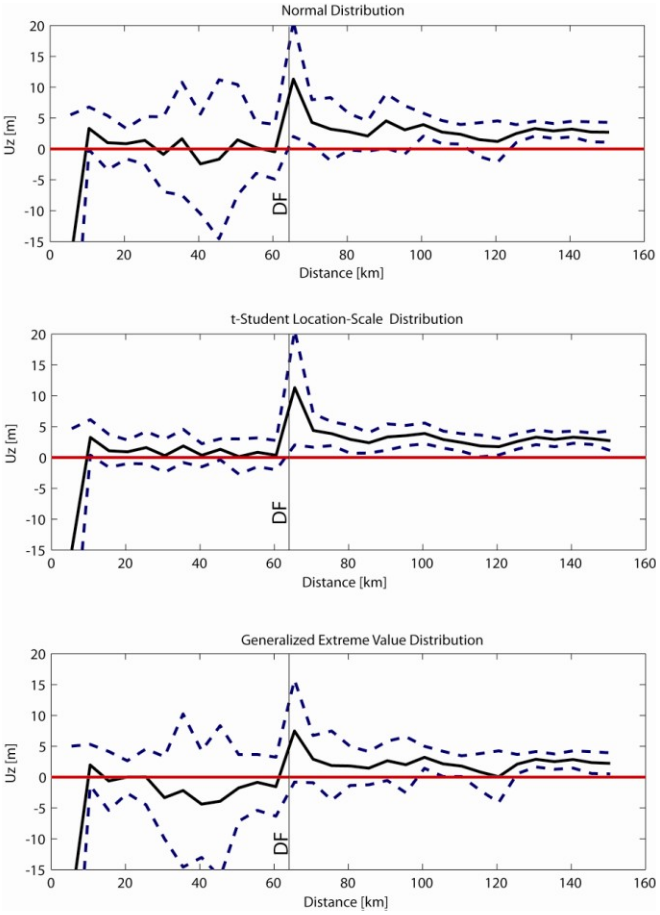

Figure S3 | Means and standard deviations of the bathymetric change, along the ILOCA track, derived from the modeled Probability Density Functions. Black line corresponds to the means along the track and the segmented blue lines show the standard deviation around the mean values. Upper panel correspond to the analysis performed with the Normal distribution, central panel shows the analysis with the t-Student Location-Scale distribution and the lower panel shows the curves obtained with the Generalized Extreme Value distribution. DF indicates the position of the deformation front.

Estimation of vertical and horizontal deformation by the differences minimization

In order to estimate the horizontal and vertical deformation during the Maule earthquake with the bathymetric data, we performed a calculation based on a simple assumption. If the shape of the seafloor remains approximately constant during the earthquake, at regional scale, the deformation measured can be seen as a horizontal (perpendicular to the trench) and a vertical displacement of the same seafloor shape, i.e, a translation without internal deformation. Therefore, if we know these horizontal and vertical displacements, we can restore the bathymetry acquired after the earthquake to its original position (before the earthquake), and then, to compare this “corrected” bathymetry with the bathymetry acquired before the earthquake. We can use, for instance, a L1 norm to compare the bathymetries obtaining a value near zero if the horizontal and vertical displacement applied are corrects.

Based on this simple criterion, we calculated both displacement values (u_x , u_z). As in the previous section, we considered only the data located in the central band of the ILOCA track (see Figure S4a. Note that the analyzed grids have a cell-size of 100 m x 100 m). The post-earthquake bathymetry were displaced all (u_x , u_z) pairs contained in the region (-25 m >= u_x <= 25 m , -25 >=u_z<= 25m), considering only integer values, i.e., the separation of values in the parametric space is 1 m. Each displaced post-earthquake bathymetry was re-gridded into the x-y coordinates of the pre-seismic bathymetry (to difference directly node by node). We then calculated the L1 norm of the differences between the post-earthquake bathymetry displaced in the space and the bathymetry before the event. By applying this procedure, the best solution for (u_x , u_z) is the value that minimize the L1 norm.

In the Figure S4, we show the results of L1 norm minimization for two segments along the ILOCA track. In both segments 68 km $< x <$ 115 km (Figure S4b and S4c) and 134 km $< x <$ 160 km (Figure S4b and S4d) we observed that the direct difference between the post and pre earthquake bathymetry is higher than zero. In the case of the western segment, if a displacement of u_x =-8 m and u_z =3 m is considering, the difference is near zero (Figure S4e), while for the eastern segment the same effect is obtained applying a displacement of u_x =-5 m and u_z =3 (Figure S4f). A negative value of u_x corresponds to a seaward displacement of the seafloor during the earthquake, and a positive value of u_z is an upward displacement of the seafloor.

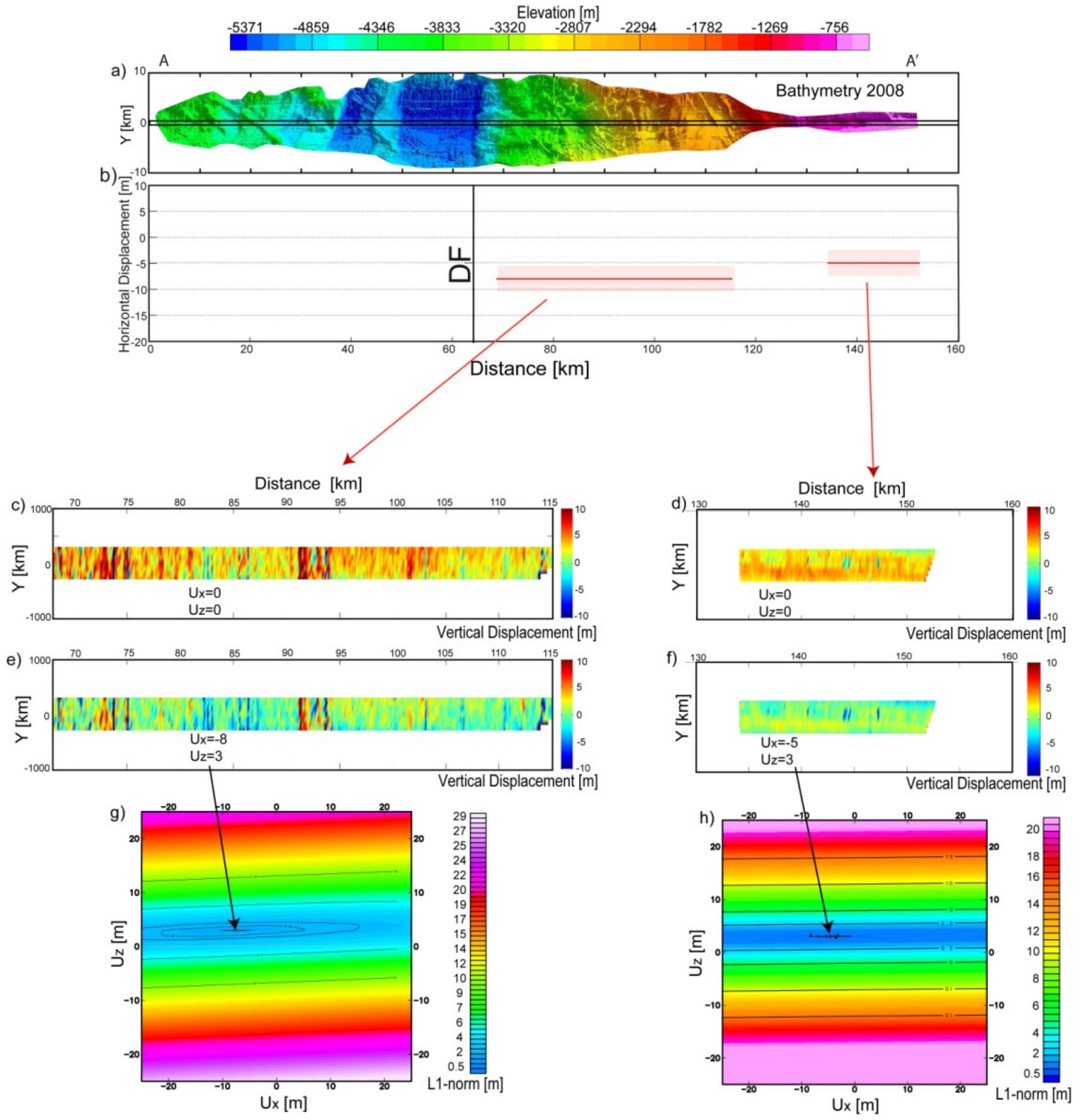

Figure S4 | Vertical and horizontal deformation by L1 norm minimization. **a**, Bathymetry along the ILOCA track acquired in 2008. The data used to estimates the deformation are located in the central band (black rectangle). **b**, Estimate of horizontal displacement in two sectors located landward from the deformation front. Negative values correspond to seaward displacement and the approximated error bars are indicated in light red. DF indicates the position of the deformation front. **c**, Direct differencing between the bathymetry after and before the Maule earthquake in the segment 68 km < x<115 km along the ILOCA track. **d**, Direct differencing between the bathymetry after and before the Maule earthquake in the segment 134 km < x<160. **e**, Bathymetry differencing in the segment 68 km < x<115 km where the bathymetry after the earthquake was moved considering displacements u_x=-8m and u_z=3m. **f**, Bathymetry differencing in the segment 134 km < x<160 km where the bathymetry after the earthquake was moved considering displacements u_x=-5m and u_z=3m. **g**, Mapping of L1-norm of the bathymetry differences as a function of horizontal and vertical displacements applied to the post-earthquake bathymetry in the segment 68 km < x<115 km. **h**, Mapping of L1-norm of the bathymetry differences as a function of horizontal and vertical displacements applied to the post-earthquake bathymetry in the segment 134 km < x<160 km.

For these two presented segments, this method gives a result coherent with the expected horizontal displacement (see main text). However, for several analyzed segments, we obtain displacements $u_x > 0$ landward from the deformation front and $u_x \neq 0$ seaward from the deformation front, which probably are erroneous values (see Figure S5a). This problem is observed in Figures S4g and S4h where the shape of L1 norm mapping is highly dependent of the u_z variation but weakly dependent of u_x, i.e., the sensitivity of the method to u_x variations is low. We expect that the lack of horizontal resolution of the bathymetry data restricted to beams just around the nadir, and the regional small slope angle of the bathymetry (which is almost null seaward from the deformation front) are the main aspects that difficult the u_x estimation. Future works based on bathymetric data with less uncertainty can give better results.

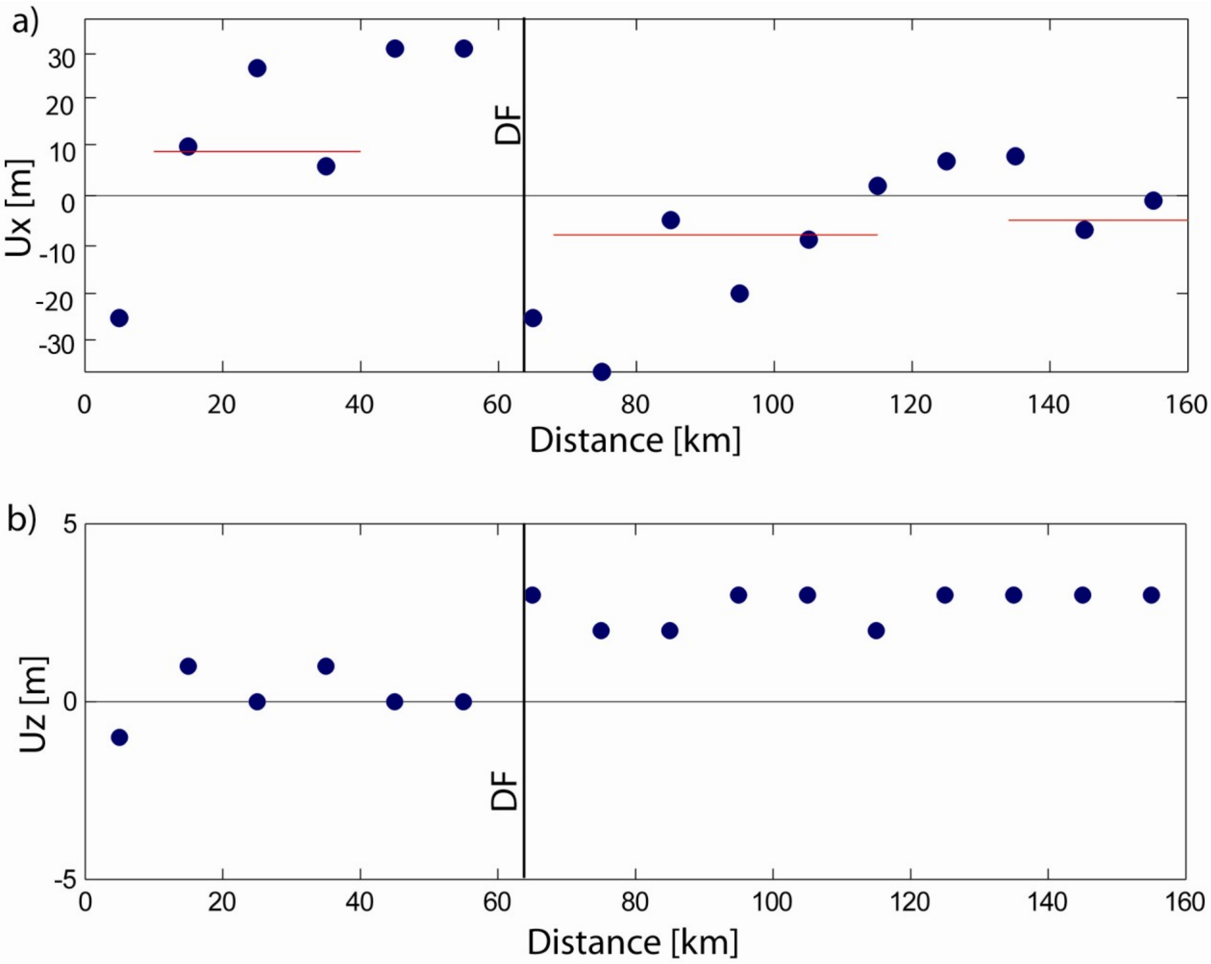

Figure S5 | Horizontal and vertical displacements (u_x , u_z) calculated in 16 segments along the ILOCA-track. **a**, Horizontal displacements, the blue dots are the values obtained in segments of 10km width and the red lines are the values obtained in the segments 10 km < x < 40 km, 68 km < x <115 km, and 134 km < x <160 km. DF indicates the position of the deformation front. **b**, Vertical displacements, the blue dots are the values obtained in segments of 10km width.

Despite the problems with the u_x estimation, it is interesting that this technique gives the clear pattern of vertical deformation u_z along the track. As it is observed in Figure S5b, if we analyze segments of the ILOCA track grids of 10 km width, the L1 norm minimization shows a clear uplift of 3 m during the earthquake, landward from the deformation front and an almost null vertical deformation seaward from the deformation front. Considering that this technique is highly dependent on u_z variations (see Figure S4), the result confirms the vertical deformation pattern obtained by the statistical analysis using a completely different approach (see the main text and previous section of this supplementary material).

Seismic data along the ILOCA-Track

The objective of this section is to show that there is no seismic evidence of large deformation inside the trench, due to the Maule earthquake. Figure S6a presents the seismic reflection line (final migrated stack) coincident to the ILOCA track that was acquired in 2012 by the R/V Melville during the expeditions of ChilePEPPER project. This seismic line was shot each 25 meters, using a source of two GI-guns in a 45/105 cm^3 configuration, and was recorded by 600 m, 48-channel streamer. As we can see, the first ~20-30 km landward from the deformation front are characterized by active accretion over a relatively thick subduction channel (~1 km thick). To the east, several forearc basins show, in general, a half-graben geometry with associated east-vergent structures and evidence of compressional deformation.

The Figure S6b shows a detailed view of the trench observed in the 2012 seismic profile (Figure S6a) where new deformation is not observed, at least at the resolution of the method. The shallow reflectors near the seafloor are continuous and unfaulted and there is no evidence of a seaward migration of the deformation front inside the trench fill. Considering the time width of the pulses in the seismic section (about ~0.005s), the maximum resolution for detection of the displacement of new faults is about 5m.

To compare the post-earthquake image with the situation before the event, Figure S5c shows a seismic profile of the trench, in the ILOCA track, acquired during the cruise JC23 of the British R/V James Cook, in March 2008. The pre-earthquake reflection seismic data have low resolution in comparison with 2012 data, because they were registered by using a 4-channel mini-streamer with an active section of 65 m. The source was a potent array of 14 Bolt airguns with a volume higher that 500 $inch^3$ each. Due some saturation and noise problems of the traces, the best representation for this data is a wiggle style seismic section (see Figure S6c). As we can see the features observed in the section are the same as those observed after the earthquake, which supports (under the resolution limitations) that the deformation did not reach the trench during the Maule earthquake.

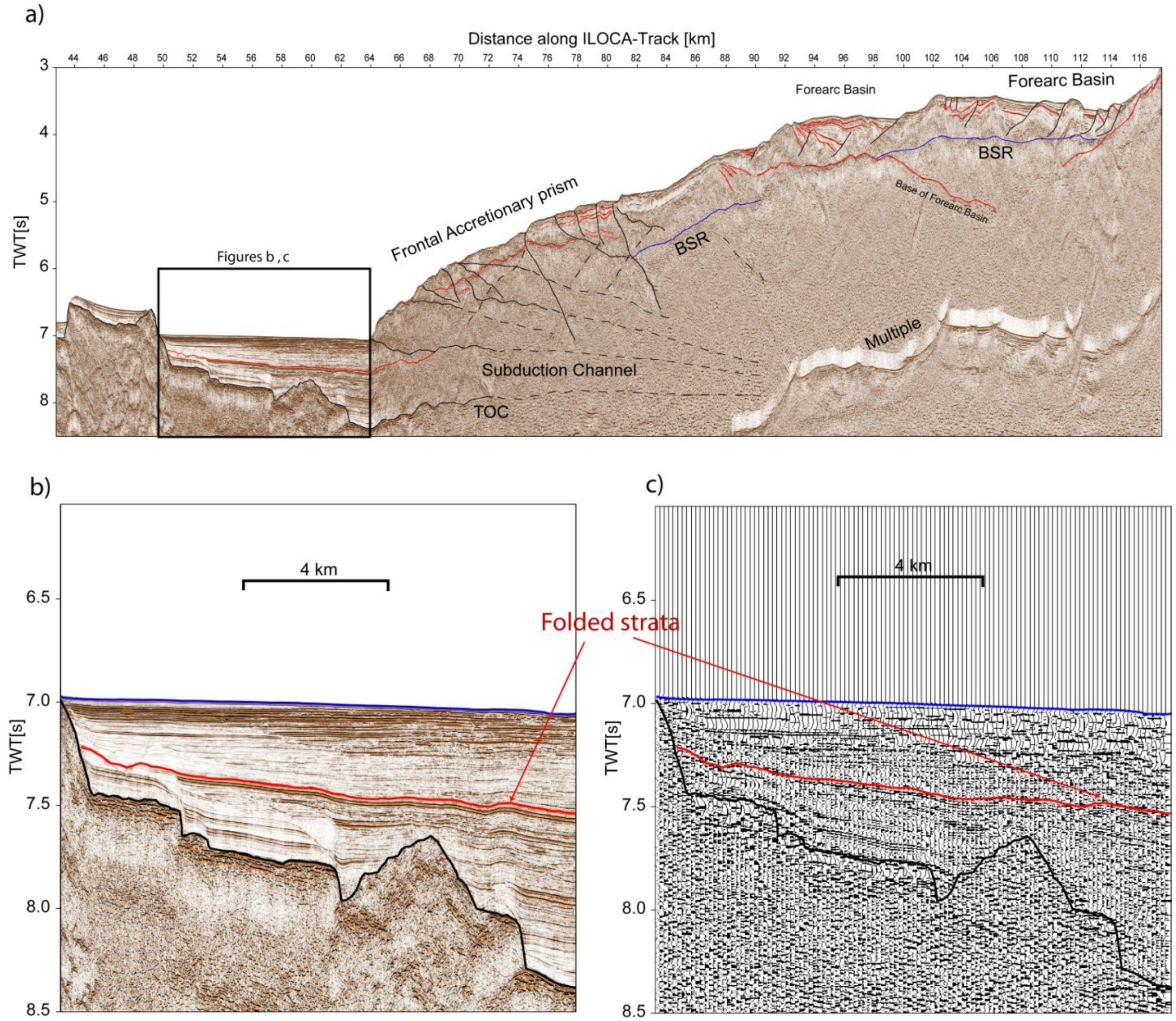

Figure S6 | Reflection seismic data along the ILOCA-track. **a**, Seismic profile acquired in 2012 by the R/V Melville (ChilePEPPER project). Tectonic domains of frontal accretionary prism and forearc basins are indicated. Some reflectors are highlighted in red to give a reference of the dip angles in different zones. The black lines and the segmented black lines indicate the main structures observed and suggested in the seismic section. **b**, A close view of the trench in the 2012 data. Blue line corresponds to the seafloor reflector, black line is the top of the oceanic crust (TOC) and red is a high amplitude reflector in the trench fill. **c**, A close view of the trench in the 2008 data acquired during the cruise JC23 of the British R/V James Cook. Blue, black and red reflectors are the same that in b.
